# Serine/Threonine Protein Kinase STK16

**DOI:** 10.3390/ijms20071760

**Published:** 2019-04-10

**Authors:** Junjun Wang, Xinmiao Ji, Juanjuan Liu, Xin Zhang

**Affiliations:** 1 High Magnetic Field Laboratory, Key Laboratory of High Magnetic Field and Ion Beam Physical Biology, Hefei Institutes of Physical Science, Chinese Academy of Sciences, Hefei 230031, China; wjunjun@mail.ustc.edu.cn (J.W.); xinmiaoji@hmfl.ac.cn (X.J.); 2 Science Island Branch of Graduate School, University of Science and Technology of China, Hefei 230026, China; 3 School of Life Sciences, Anhui University, Hefei 230601, China; 4 Institutes of Physical Science and Information Technology, Anhui University, Hefei 230601, China

**Keywords:** STK16, kinase, fatty acylation, phosphorylation, Golgi apparatus, protein secretion and sorting, cell cycle

## Abstract

STK16 (Ser/Thr kinase 16, also known as Krct/PKL12/MPSK1/TSF-1) is a myristoylated and palmitoylated Ser/Thr protein kinase that is ubiquitously expressed and conserved among all eukaryotes. STK16 is distantly related to the other kinases and belongs to the NAK kinase family that has an atypical activation loop architecture. As a membrane-associated protein that is primarily localized to the Golgi, STK16 has been shown to participate in the TGF-β signaling pathway, TGN protein secretion and sorting, as well as cell cycle and Golgi assembly regulation. This review aims to provide a comprehensive summary of the progress made in recent research about STK16, ranging from its distribution, molecular characterization, post-translational modification (fatty acylation and phosphorylation), interactors (GlcNAcK/DRG1/MAL2/Actin/WDR1), and related functions. As a relatively underexplored kinase, more studies are encouraged to unravel its regulation mechanisms and cellular functions.

## 1. Introduction

The human protein kinases belong to a large family that consists of more than 500 members [1,2]. They are involved in the regulation of many different cellular processes, such as cell growth, survival, proliferation, apoptosis, and metabolism [3,4,5,6]. These kinases have homologous catalytic domains that consist of 250–300 amino acid residues [7,8]. Polymerase chain reactions using primers derived from the conserved sequences were used to amplify new kinase families [9,10]. In this way, Ligos et al. were the first to identify STK16 (Ser/Thr kinase 16), a member of a new subfamily of Ser/Thr kinases, which was named PKL12 (protein kinase expressed in day 12 fetal liver), from mouse fetal liver (E12) [11]. It was also known as Krct (kinase related to cerevisiae and thaliana) [12], EDPK (embryo-derived protein kinase) [13], MPSK (myristoylated and palmitoylated Ser/Thr protein kinase) [14] and TSF-1 (TGF-β stimulated factor 1) [15] due to its diverse discovery experiences, but conserved among vertebrates. A series of studies on the characterization and biological function analysis of this kinase have gradually revealed that it is actually a novel member of the NAK (Numb-associated family of protein kinases) kinase family with unique features and functions [16,17]. Although twenty years have passed since the discovery of STK16, it is still a relatively underexplored kinase. However, recent studies have revealed its important roles in cell signaling, cell cycle, Golgi assembly regulation, and protein secretion and sorting. This review aims to provide a comprehensive review about the STK16 molecular structure, expression, post-translational modification, interactors/substrates, and its cellular functions.

## 2. Distribution and Characterization of STK16

### 2.1. Distribution

STK16 is widely distributed and its homologues exist in eukaryotes from yeasts to humans, although most studies focused on the human and mice homologues. Moreover, *stk16* was identified on chromosome 1 in mice from Mouse Genome Informatics (http://www.informatics.jax.org) but localized to human chromosome 2 according to the Gene Ontology Annotation (GOA) database (https://www.ebi.ac.uk/GOA). Based on in situ hybridization, Northern blot, and RT-PCR, it was found that STK16 was widely expressed throughout murine development and in adult tissues on the mRNA level [11,12,13,14,15]. In addition, although having a ubiquitous distribution, STK16 is expressed preferentially and specifically within multiple tissues, in particular within the liver, kidney, and testis [11,12,13,15]. Furthermore, STK16 expression within the same tissue is higher in certain cell types. For example, compared with the mesenchymal compartments, STK16 expression level was higher in epithelial compartments [12,18]. Western blot results showed that STK16 was expressed in various cell lines, but it preferred to be expressed in adherent cells compared with suspension cells [19]. In cells, STK16 mainly localizes to the Golgi [11,19,20,21] and also enters the nucleus under certain circumstances [19].

### 2.2. Molecular Characterization of STK16 

STK16 homologues in various species share different similarity. Amino acid alignment results showed that the sequence similarity was greater than 90% in different vertebrates but only 29% to the *Saccharomyces cerevisiae* STK16 homologue named ENV7 [22]. The ORF (open reading frame) of mammalian STK16 contains 915 nucleotides and encodes 305 amino acids. It comprises a complete kinase catalytic domain, a very short N-terminal domain, and a brief C-terminus. Alignment of the kinase domain of STK16 with others illustrated that it is a new Ser/Thr kinase, displaying a conservative sequence element of Ser/Thr kinase, but lacking the necessary amino acids in subdomain VIb and VIII that are conserved in tyrosine kinases [11,12,13,14]. Activation segment sequences analysis together with secondary structure prediction suggested that it is distantly related to the other kinases and belongs to the family of human NAK [16]. According to the classification and phylogenetic analysis of Human Kinome, the NAK family does not belong to any group [2,23]. In fact, the crystal structure of STK16 was solved by Eswaran et al. in 2008 revealing the atypical activated loop ASCH (activation segment C terminal helix) in its catalytic domain, which also proved this classification [17].

**Figure 1:**
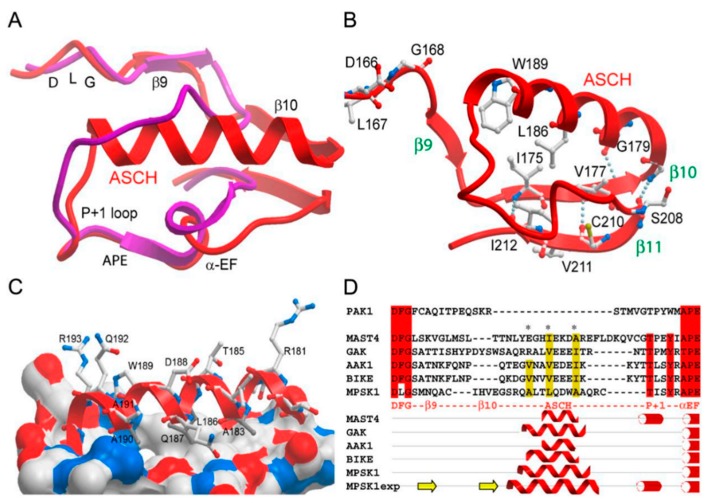
STK16 activation loop architecture [17]. (**A**) Structural overlay of the activation loop of active Aurora A (PDB ID code: 1OL7) (shown in magenta) with MPSK1 (shown in red). The main structural elements are labeled. DLG motif (Asp-Leu-Gly) is the initiation of the activation segment of STK16. APE motif (Ala-Pro-Glu) is the end of the activation segment of STK16 and P + 1 loop is before of the APE motif. (**B**) Hydrogen bonds and hydrophobic interactions stabilizing the activation segment of MPSK1 are shown as dotted lines and the residues involved in stabilization are shown in ball-and-stick representation. The interacting β sheet (β11), the P + 1 loop, and the ASCH, as well as the helix αEF, are labeled. (**C**) Interface of the ASCH interacting with the lower kinase lobe. Hydrophobic residues are indicated as solid white surfaces. (**D**) Prediction of similar activation loop helices present in the kinome. Secondary structure elements predicted to be smaller than three residues have been deleted. The experimentally determined secondary structure (MPSK1exp) and the predicted one of MPSK1 are also shown. The activation segment helix and helix αEF were predicted accurately, whereas the β sheet secondary structure was not recognized by the prediction program. One representative member of the MAST kinase family predicted to contain an activation loop helix is also shown. Hydrophobic residues, buried in the interface between the ASCH and the lower kinase lobe, are indicated (^∗^) in the sequence alignment. The ninth and tenth β sheets are indicated by yellow arrows. ASCH is indicated by red wavy line. P + 1 loop and helix αEF are indicated by red bar with white circle. Figure was adapted from ref [17].

Besides STK16, the human NAK family also includes AAK1 (adaptor-associated kinase 1), BIKE/BMP2K (BMP-2-inducible kinase), and GAK (cyclin G-associated kinase). Though their catalytic domains sequences have less than 30% similarity and share almost no conservation outside the kinase domain [16,17], their crystal structures solved by the Stefan Knapp group showed an ASCH architecture in all human NAKs (Figure 1) [17]. It provides new ideas and insights for a comprehensive understanding of the molecular characteristics of NAK family proteins and their related physiological functions. The assembly of the regulation region independent of atypical activation loop phosphorylation explains why NAKs are constitutively active. Moreover, the variable substrate binding grooves of NAKs suggest that they could participate in a broad range of cellular functions through interacting with different substrates. 

Among the kinases in the NAK family, STK16 is the less studied one. However, since the NAK kinases share the same distinctive ASCH structure, studies about the other family members can help us to understand the function of STK16. For example, in Drosophila, NAK interacts with the phosphotyrosine binding domain (PTB) of Numb, regulates asymmetric cell division and confers distinct fates to daughter cells [24,25,26]. In humans, AAK1 binds directly to adaptin, the membrane-tethered active form of Notch, or Numb, to regulate a variety of activities including clathrin-mediated endocytosis [27,28], Notch signaling pathway [29], or coated pit maturation [30]. Another member BIKE/BMP2K was found to play an important regulatory role in endocytosis associated with Numb [31]. Similarly, GAK, interacting with cyclin G-CDK5, is a potential regulator of clathrin-mediated membrane trafficking and mediates binding of clathrin and adaptors to the plasma membrane and the trans-Golgi network [32,33]. NAKs have been implicated in many diseases and supposed to be potential drug targets. For example, inhibition of AAK1 kinase has become a novel therapeutic approach to treat neuropathic pain, schizophrenia, Parkinson’s disease, and hepatitis C virus infection [34,35,36]. GAK has been discussed as a potential drug target for the treatment of viral infections due to its involvement in entry and production of multiple viruses [36,37,38]. Furthermore, BIKE was reported as a cellular factor associated with HIV-1 replication [39]. Recently, STK16 was also found to participate in cell division, cell signaling, and protein secretion and sorting, which will be discussed in detail later. STK16 IN-1, a small molecule inhibitor with high selectivity, has been developed to specifically inhibit the kinase activity of STK16 [40]. STK16-IN-1 could reduce cancer cell numbers and potentiate the antiproliferative effects of some chemotherapeutics [40]. Thus, inhibition of STK16 is expected to be developed as a novel therapeutic approach for cancers.

## 3. Posttranslational Modification of STK16 

### 3.1. STK16 Undergoes Specific Fatty Acylation Modification

Many protein kinases can be modified by myristic acid or palmitic acid to regulate fundamental biological processes of mammalian cells [41,42,43,44]. Myristoylation occurs in the cytosol, where the 14-carbon saturated fatty acid myristate is linked to an N-terminal Gly of the protein by a stable amide bond. Cys-palmitoylation, also known as *S*-palmitoylation, the 16-carbon saturated fatty acid palmitate is covalently linked to one or more specific Cys residues on the side chain via unstable thioester bonds [45,46]. Although both modifications could usually guide proteins to bind membrane, only the palmitoylation is reversible, which indicates that it could dynamically regulate kinase functions [47]. The best example is that Ras proteins are directed to the correct intracellular organelles for trafficking and perform activity just by palmitoylation [48]. In addition, it is reported that palmitoylation of three cysteines at the C-terminus of GRK6 directs its membrane binding and further regulates its subcellular distribution [49]. In vivo, both fatty acylations are usually coordinated and jointly regulate protein localization and function. Myristoylation first occurs at the N- terminal amino acids and this enables the protein to approach the membrane-bound palmitoyl acyltransferases and gets palmitoylated. Src family kinases [50,51] and AKAPs (A-kinase anchoring proteins) have been identified to be acylated by this way [52]. Another example is CDPK, a rice calcium-dependent protein kinase, whose myristoylation is essential for membrane localization, and palmitoylation is requisite for its full association [53].

**Figure 2:**
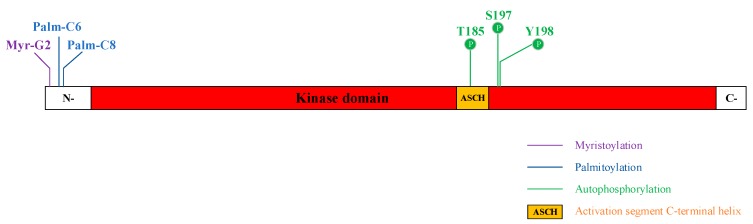
Illustration of posttranslational modification of STK16.

STK16 may regulate its subcellular localization through fatty acylation modification. STK16 have a Gly at the position 2 and Cys at the position 6 and 8 of the N-terminus, respectively (Figure 2). These three conserved amino acids could be acylated by myristic acid and palmitic acid. In G2A (Gly 2 to Ala) mutation, myristoylation and palmitoylation were abolished. However, in C6,8S (Cys 6 and 8 to Ser) mutation, myristoylation was still detected but palmitoylation was completely lost. Therefore, it is suggested that the myristoylation of Gly2 in STK16 is necessary for the palmitoylation of Cys6 and Cys8 in this protein [14]. The homologue ENV7 in S.cerevisiae also has conservative palmitoylation sites at the N-terminus. Mutations at these sites result in palmitoylation abolishment and altered subcellular localization [22,54]. Besides, further analysis revealed that these acylation sites of ENV7 were not redundant and function in regulating ENV7′s stability, localization, phosphorylation, and vacuolar fusion, respectively [55]. Although this evidence indicates that ENV7 may perform different physiology functions through fatty acylation, the fatty acylation regulation of mammal STK16 homologue is still unknown.

### 3.2. STK16 is a Constitutively Active Ser/Thr Protein Kinase

In vitro, STK16 is capable of both autophosphorylation and phosphorylation of other substrates, such as MBP (myelin basic protein), Histone H1, PHAS-1, enolase, and Elk [11,12,13,14,15]. Phospho-amino acid analysis showed that the autophosphorylation sites of STK16 were mainly on Thr [14], whereas substrates of STK16 are phosphorylated mainly at Thr and Ser [11,13,14]. Screening a peptide library, an optimal substrate sequence of X-X-P/V/I-Φ-H/Y-T*-N/G-X-X-X (where Φ is an aliphatic residue, and * stands for the phosphorylated residue) was determined for STK16 [17]. This is consistent with the results of crystal analysis of STK16 [17]. Moreover, tagged or untagged STK16 protein, such as GST-, His-, and V5-tag, whether expressed and purified by the prokaryotic system (*Escherichia coli*) or the eukaryotic system (yeast, Sf9, Cos-1, or IP (immunoprecipitation) products from COS-7 cells transfected with STK16 cDNA), are all active kinase. The only two kinase-dead mutants generated in previous studies are K49M (Lys49 is in the ATP-binding site of STK16) and E202A (Glu202 is a key site to maintain the activation segment of kinase) [15,21,56].

In many protein kinases, phosphorylation on the Ser, Thr, or Tyr residues in the activation loop is required. The negatively charged phosphate groups compensate for the high positive charge in the activation segment, and the catalytic loop HRD (His-Arg-Asp residues in the catalytic loop) motif maintains the stability of the activation segment through polar interaction. Thus, activation loop phosphorylation is the most common method for regulating kinase activity [56,57]. Interestingly, the HRD arginine (Arg147) in STK16 forms a large number of polar interactions with the residues of the activation segment. In addition, a cluster of hydrophobic residues are close and stabilize the activation loop. Therefore, STK16 has a well-ordered catalytic conformation of the activation loop without dependence on phosphorylation or exogenous induction and is constitutively active (Figure 1) [17]. In fact, many kinases become constitutively active to be involved in the specific signaling pathway. For example, Akt becomes constitutively active when directed to the membrane by myristoylation, and this change induces glucose uptake into adipocytes in the absence of insulin and directs lipid synthesis [58]. Moreover, NIK (NF-κB-inducing kinase) is usually present as an autoinhibited form, and its constitutively active kinase domain is blocked by the inhibitory element [59]. After cytokine induction, NIK undergoes conformational changes and regulates NF-κB signal transduction through constitutive kinase activity [60]. However, the hyperactive NIK leads to autoimmune disease and cancer [61].

STK16 is capable of autophosphorylation at Thr185, Ser197, and Tyr198 of the activation segment (Figure 2). In the structure of unphosphorylated STK16, Ser197 and Tyr198 are buried in the hydrophobic cleft, resulting in slow phosphorylation of these two sites [17]. Besides these sites, there are also other potential phosphorylation sites in the activation loop of STK16, including Ser169, Ser180, and Thr95, which has not been investigated yet. Since Ser/Thr kinases usually possess multiple phosphorylation residues on the activation loop that have distinct effects on autophosphorylation [62,63,64], whether these sites are involved in the STK16 autophosphorylation and kinase activity needs further investigations.

### 3.3. The Relationship between Fatty Acylation and Phosphorylation of STK16

Many protein kinases are cooperatively regulated by fatty acylation and phosphorylation. For example, palmitoylated GRK6 has significantly increased kinase activity more than nonpalmitoylated wild-type GRK6 and a nonpalmitoylatable mutant GRK6 [65]. The viral protein TLCYnV C4 (Tomato leaf curl Yunnan virus C4) shuttles between the nucleus and the cytoplasm. Phosphorylated TLCYnV C4 promotes its myristoylation, then C4 achieve nuclear export [66]. For MARCKS (myristoylated alanine-rich protein kinase C substrate), myristoylation is the basis for its membrane localization. However, its phosphorylation by PKC promotes its rapid dissociation [67,68]. The mutations of all three cysteines (Cys13, 14, 15) or two of them at the N-terminal Env7, the yeast homologue of STK16, not only cause changes in membrane localization, but also affect its kinase activity by significantly decreasing the autophosphorylation level [54,55]. All this evidence suggests that there is some interplay between the phosphorylation and fatty acylation of these kinases. The relationship between the phosphorylation and fatty acylation of mammalian STK16 is currently unknown.

## 4. Cellular Functions of STK16 

There are a few studies indicating that STK16 binds different interactors (GlcNAck/DRG1/MAL2/WDR1/Actin) and participates in various physiological activities, including the TGF-β signaling pathway and TGN protein secretion and sorting, as well as cell cycle and Golgi assembly regulation.

### 4.1. STK16 Participates in the TGF- β Signaling Pathway

TGF-β signaling pathways include both Smads-dependent and Smads-independent pathways. Due to the diversity of TGF-βs, TGF-β family receptors, Smads, and DNA-elements, as well as the different interaction patterns between various proteins [69,70,71,72], TGF-β signaling pathways can induce very diverse physiological and pathological responses [73,74,75,76], including immunity [77], cancer [78], fibrosis [79], and hematopoietic homeostasis [80]. 

It was reported that STK16 possesses DNA-binding ability independent of its kinase activity. It binds to GC-rich elements of TGF-β responsive CNP (C-type natriuretic peptide) promoter and VEGF (vascular endothelial growth factor) promoter to activate them [15]. Moreover, as well as the mRNA and protein levels of STK16 responding to TGF-β treatment, elevated STK16 could also increase the mRNA level of STK16, which indicates that there may be a positive feedback loop between the transcriptional activity of STK16 and its own protein level [19]. 

It was reported that STK16 is able to enter the nucleus. After Golgi disorganization by treatment with BFA or nocodazole, STK16 lost the Golgi localization and translocated into the nucleus in NIH3T3 cells in a kinase activity independent way [19]. In HT1080 cells, the kinase-dead mutant STK16-E202A can also induce the expression of VEGF, which is consistent with the fact that STK16 activates VEGF gene promoter transcriptional activity independently of its kinase activity [15]. Moreover, higher STK16 transcriptional activity was induced by BFA or nocodazole treatment in STK16 wild type and E202A overexpressed cells, which is consistent with previous research that STK16 nuclear translocation could up-regulate STK16 transcription [15]. In fact, the alternative splicing of STK16 is related to the differential transcription activity of ELK1 [81]. The change in the exon inclusion ratio of STK16 was related to ELK1 transcription, which mainly depends on the fourth exon located in the kinase domain.

In conclusion, STK16 may be a nuclear factor that enters the nucleus following certain stimuli, participating in the TGF-β signaling pathway and regulating transcriptional activity. However, very low nuclear localization of endogenous STK16 has been detected in unstimulated cells, which may be caused by the formation of complexes between STK16 and other proteins. In addition, the nuclear localization between STK16 in various species are also likely different. Moreover, most current commercial STK16 antibodies have limited specificity or application, and development of more specific STK16 antibodies is crucial for further investigations of STK16.

### 4.2. STK16 and GlcNAcK (N-Acetylglucosamine Kinase)

GlcNAc (N-Acetylglucosamine) is an important component of bacterial cell wall peptidoglycan [82], fungal cell wall chitin [83], and the extracellular matrix of animal cells [84]. In animal cells, GlcNAc also regulates the distribution of glycoproteins at the cell surface through glycosylation and further regulates cell signaling transduction [85,86]. Mammalian GlcNAcK (N-Acetylglucosamine Kinase) converts GlcNAc from lysosomal degradations or nutritional sources to GlcNAc-6-phosphate. Eventually, GlcNAc-6-phosphate can enter the catabolic pathway to form fructose 6-phosphate [87] or enter the anabolic pathway to synthesize UDP-GlcNAc [88]. Moreover, UDP-GlcNAc serves as a donor for various glycoconjugates and glycans to provide GlcNAc [89,90]. The metabolic imbalance of GlcNAc causes serious damage to cells [91,92]. In addition, GlcNAcK also has some non-traditional functions. It is highly expressed in neurons [93], and the interaction of GlcNAcK-Dynein-Golgi could regulate the growth of axons [94] and dendrites [95] of neurons. Moreover, the interaction of GlcNAcK-Dynein-Lis1-NudE1 plays an important role in the prophase and metaphase of mitosis [96]. Meanwhile, GlcNAcK also has different subnuclear distribution [97]. 

GlcNAcK was reported as another STK16 interactor [20], although GlcNAcK is not a substrate of STK16, and STK16 does not affect GlcNAcK activity in vivo or in vitro. Although GlcNAcK is unable to regulate STK16 autophosphorylation, it greatly affects the phosphorylation effect of STK16 on its substrates. In the interphase NIH-3T3 cells, endogenous GlcNAcK is located predominantly in the perinuclear area and the cell periphery. Endogenous STK16 is associated mainly with the Golgi, co-localizing partially with GlcNAcK. However, when GlcNAcK was overexpressed with STK16, they highly colocalized in a vesicular pattern. 

The STK16/GlcNAcK interaction pattern unravels their new function of translocation. GlcNAcK regulates the Golgi complex transport by the dynein motor [95]. Therefore, as an interactor of the dynein complex, GlcNAcK may be involved in the transportation of STK16. In particular, STK16 is a Golgi resident protein [11,19,20,21]. The interaction between STK16 and GlcNAcK causes the redistribution of GlcNAcK and may alter the metabolic balance of GlcNAc, thus participating in glucose metabolism. It is also possible that STK16 plays a role in the non-traditional functions of GlcNAcK, which needs further investigation.

### 4.3. STK16 and MAL2/WDR1 (Myelin and Lymphocyte Protein 2/ WD Repeat Containing Protein-1)

MAL2, a raft protein of the MAL proteolipid family, contains four transmembrane domains and regulates the transcytotic delivery pathways of various proteins [98]. It is necessary for basolateral-to-apical transcytosis in HepG2 cells, delivering membrane-associated proteins and exogenous cargo, such as pIgA (polymeric immunoglobulin A-receptor) and glycosylphosphatidylinositol-anchored protein CD59 [99,100,101]. The interaction between STK16 and MAL2, discovered by split-ubiquitin yeast two-hybrid assays, regulates secretory soluble cargo sorting into the constitutive secretory pathway at the TGN (trans-Golgi network) in hepatocytes [102]. Overexpression of STK16 kinase-dead mutant E202A or knockdown of MAL2 in polarized hepatic WIF-B cells impairs soluble cargo secretion, resulting in decreased secretion of albumin and haptoglobin. The synthesis and process of albumin in E202A-overexpressing cells or MAL2-knockdown cells are not interfered, but they are not secreted as in wild-type cells. Instead, they are directly degraded in lysosomes after being delivered from ER to the Golgi. Therefore, albumin detection was recovered in E202A-overexpressing cells or MAL2-knockdown cells after lysosomal deacidification. Thus, STK16 and MAL2 may play a key role in protein sorting at the TGN. 

Consistent with the interaction between STK16 and MAL2, several proteins interact with MAL2 to influence secretion. For example, Formin INF2, associating with cdc42 and MAL2, regulates basolateral-to-apical transcytosis and lateral lumen formation in HepG2 cells [103]. In addition, MAL2 controls vesicle transportation through interaction with TPD52 (tumor protein 52)-like proteins [98]. In Candida albicans, STK16 homologue named CaENV7 interacts with two TGN-resident proteins Imh1p and Arl1p. Imh1 is phosphorylated by CaEnv7 and their interaction affects the localization of Imh1, thus maintaining the morphology and function of TGN and having an influence on protein secretion and delivery [104]. Moreover, the overexpression of STK16 leads to abnormal endbud formation in the mammary gland during puberty, which may be caused by abnormal cytokine secretion, and STK16 may regulate the process [18]. In a word, STK16, highly expressed in the liver with constitutive kinase activity, regulates the secretory function through the interaction with MAL2. 

Furthermore, increased E202A levels further impaired albumin secretion, suggesting that STK16 kinase activity is required for soluble secretion [102]. Recently, WDR1 was reported as a possible substrate of STK16, regulating constitutive secretion [105]. WDR1, referred to as AIP1 (actin-interacting protein 1), was first identified as an actin-binding protein. As the binding site of actin and cofilin, its conserved WD40 repeat sequences regulate the morphology and function of the cytoskeleton [106,107,108], and the hepatic secretion requires actin remodeling [109,110].

Together with MAL2 and WDR1, it is likely that STK16 regulates the constitutive secretion in the hepatocyte. Although STK16 interacts with MAL2 and is a candidate kinase for WDR1, further studies are necessary to confirm whether MAL2 is the substrate of STK16 and whether WDR1 is the direct substrate. 

### 4.4. STK16 and DRG1 (Developmentally Regulated GTP Binding Protein 1)

DRG1 (developmentally regulated GTP-binding protein 1) belongs to the DRG family that contains DRG1 and DRG2 [111], which is significantly expressed in the embryonic brain and downregulated during development [112,113]. DRG1 is highly expressed in various embryonic tissues but is greatly decreased in newborn animals [112]. In addition, in mouse and human, DRG1 specifically binds to SCL/TAL (stem cell leukemia/T-cell acute lymphoblastic leukemia (T-ALL) 1), a highly conserved basic helix-loop-helix transcription factor participating in cell growth and differentiation and required for normal hematopoietic development [114,115]. 

Evidence from pull-down experiments has shown the specific interaction between DRG1 and STK16 [17]. Furthermore, the 65 amino acids at the N-terminus of DRG1 are sufficient for STK16 binding, and DRG1 is phosphorylated at Thr100 within the GTPase domain by STK16. In contrast, DRG2, which has a Cys100 instead of Thr, does not interact with STK16 [17]. Similarly, although DRG1 and DRG2 are both widely expressed in human and mouse tissues and their sequences share 62% identity [111], they function differently by binding distinct regulatory proteins. For instance, DFRPs (DRG family of regulatory proteins) maintain the stability of DRGs through specific binding, for example, DFRP1 binds DRG1, and DFRP2 binds DRG2, respectively [111]. In other species, the transcription or stability of DRG1 and DRG2 transcripts were also found to be regulated differently [116]. Moreover, DRG1 was identified as a direct microtubule binding protein, which drives microtubules into bundles and stabilizes microtubules. In DRG1-knockdown cells, the progression from prophase to anaphase is delayed and spindle formation is slowed down [117], whereas DRG2 interacts with tau as a mechanism for regulating microtubule dynamics in HeLa cells [118].

These observations indicate that DRG1 is a specific interactor and substrate of STK16. However, further experiments are needed to reveal the mechanism by which STK16 regulates DRG1 signaling and its related cellular functions.

### 4.5. STK16 and Actin

**Figure 3:**
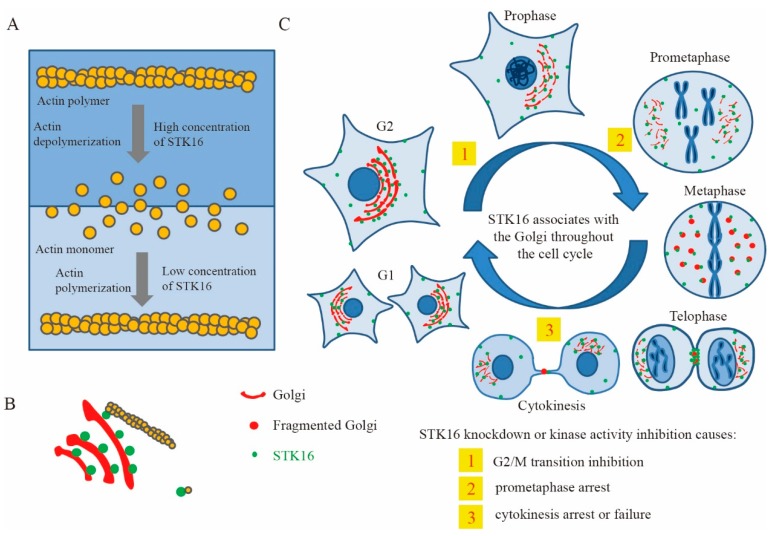
The model of STK16 functions in actin, the Golgi, and cell cycle regulation [21]. (**A**) STK16 kinase is a novel actin binding protein that regulates actin polymerization and depolymerization in a concentration-dependent manner. (**B**) STK16 localizes to the Golgi and bridges the Golgi with actin. (**C**) STK16 plays important roles in G2/M transition and mitotic progression as well as cytokinesis. Figure was adapted from [21].

Previous studies showed that STK16 can interact with actin to regulate the Golgi apparatus dynamics. Although the interaction between STK16 and actin is independent of its kinase activity, STK16 regulates actin dynamics in a concentration and kinase activity-dependent way (Figure 3) [21]. 

As an important membranous organelle of eukaryotes, Golgi apparatus is a central component of the endomembrane system and plays key roles in trafficking, post-translational modifications of proteins, and lipid biosynthesis [119]. It has been widely reported that actin and actin-binding proteins that localize on the Golgi apparatus could regulate the structure and function of the Golgi [120,121]. For example, GRASP65 (Golgi reassembly and stacking proteins 65), distributed on the peripheral membrane of the Golgi apparatus, interacts with the actin-binding protein Mena and leads to its own homologous oligomerization, which mediates the stacking of the Golgi cisternae and ribbon formation [122,123,124]. Furthermore, GRASP55 and GRASP65 are phosphorylated to be depolymerized and control Golgi fragmentation in mitosis [125,126]. During the highly dynamic and precisely regulated mitosis, the Golgi structure experiences fragmentation and remodeling, which is critical for mitotic progression [127]. Therefore, STK16 knockdown or kinase inhibition could arrest cells in prometaphase and cytokinesis and delay mitotic progressions [21,40]. 

Besides actin and actin-binding proteins, the assembly, morphology, localization, and orientation of the Golgi apparatus are also correlated to microtubules and microtubule-related proteins [128,129,130], and the regulation between cytoskeleton with the Golgi complex has been a hot topic for mitotic regulation studies [131]. STK16 interacts with cytoskeleton to regulate the Golgi structure, whereas the detailed regulatory mechanisms are unexplored, such as the binding mechanism of STK16 and actin, which step of Golgi apparatus assembly STK16 and actin are involved in, and how STK16 and actin regulate the Golgi assembly. The elucidation of these questions will contribute to our understanding of the regulation between the cytoskeleton and the Golgi apparatus.

## 5. Conclusions and Perspectives

**Figure 4:**
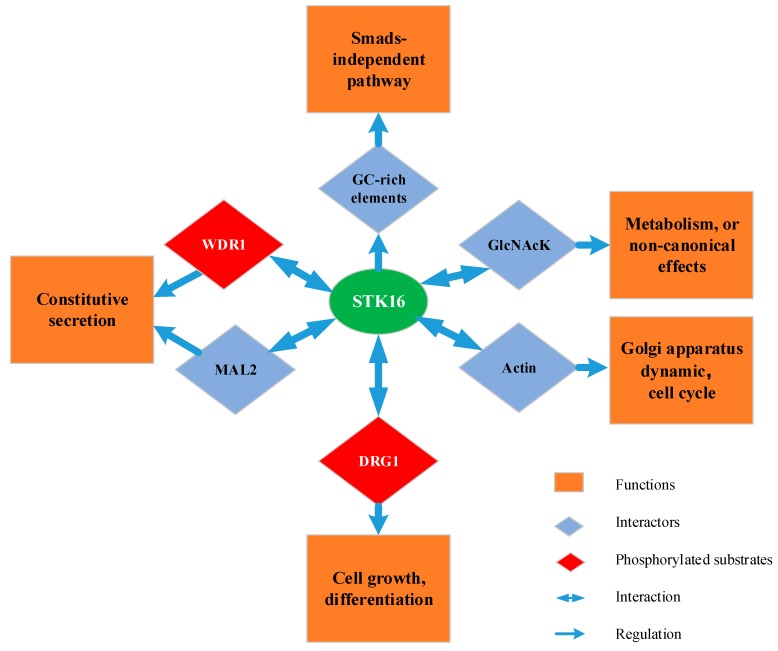
STK16 pathways.

As a membrane-associated protein with constitutive kinase activity, STK16 has been found to participate in various signaling pathways and cellular process regulations in eukaryotes, such as TGF-β signaling pathway, TGN protein secretion and sorting, as well as the Golgi assembly and cell cycle (Figure 4). Moreover, we also know that STK16 undergoes at least two post-translational modifications, fatty acylation, and phosphorylation. However, what serves as STK16 kinase activity switch, what the upstream regulators and kinases are, and what the specific localization of different forms of phosphorylated STK16 during mitosis are all unclear so far, which encourages people to conduct further mechanistic studies to unravel its cellular functions and regulation mechanisms so that we can have a more comprehensive understanding about this kinase.

## References

[1] Wilson L.J., Linley A., Hammond D.E., Hood F.E., Coulson J.M., Macewan D.J., Ross S.J., Slupsky J.R., Smith P.D., Eyers P.A. (2018). New Perspectives, Opportunities, and Challenges in Exploring the Human Protein Kinome. Cancer Res..

[2] Manning G., Whyte D.B., Martinez R., Hunter T., Sudarsanam S. (2002). The protein kinase complement of the human genome. Science.

[3] Gulluni F., De Santis M.C., Margaria J.P., Martini M., Hirsch E. (2019). Class II PI3K Functions in Cell Biology and Disease. Trends Cell Biol..

[4] Fu J., Bian M., Jiang Q., Zhang C. (2007). Roles of Aurora Kinases in Mitosis and Tumorigenesis. Mol. Cancer Res..

[5] Sui X., Kong N., Ye L., Han W., Zhou J., Zhang Q., He C., Pan H. (2014). p38 and JNK MAPK pathways control the balance of apoptosis and autophagy in response to chemotherapeutic agents. Cancer Lett..

[6] García Z., Kumar A., Marques M., Cortes I., Carrera A.C. (2006). Phosphoinositide 3-kinase controls early and late events in mammalian cell division. Embo J..

[7] Hanks S.K., Quinn A.M. (1991). Protein kinase catalytic domain sequence database: Identification of conserved features of primary structure and classification of family members. Methods Enzymol..

[8] Hanks S.K., Quinn A.M., Hunter T. (1988). The protein kinase family: Conserved features and deduced phylogeny of the catalytic domains. Science.

[9] AbdElsalam K.A. (2003). Bioinformatic tools and guideline for PCR primer design. Afr. J. Biotechnol..

[10] Bachman J. (2013). Reverse-transcription PCR (RT-PCR). Methods Enzymol..

[11] Ligos J.M., Gerwin N., Fernández P., Gutierrez-Ramos J.C., Bernad A. (1998). Cloning, Expression Analysis, and Functional Characterization of PKL12, a Member of a New Subfamily of ser/thr Kinases. Biochem. Biophys. Res. Commun..

[12] Stairs D.B., Gardner H.P., Ha S.I., Copeland N.G., Gilbert D.J., Jenkins N.A., Chodosh L.A. (1998). Cloning and characterization of Krct, a member of a novel subfamily of serine/threonine kinases. Hum. Mol. Genet..

[13] Kurioka K., Nakagawa K., Denda K., Miyazawa K., Kitamura N. (1998). Molecular cloning and characterization of a novel protein serine/threonine kinase highly expressed in mouse embryo 1. Biochim. Biophys. Acta.

[14] Berson A.E., Young C., Morrison S.L., Fujii G.H., Sheung J., Wu B., Bolen J.B., Burkhardt A.L. (1999). Identification and characterization of a myristylated and palmitylated serine/threonine protein kinase. Biochem. Biophys. Res. Commun..

[15] Ohta S., Takeuchi M., Deguchi M., Tsuji T., Gahara Y., Nagata K. (2000). A novel transcriptional factor with Ser/Thr kinase activity involved in the transforming growth factor (TGF)-beta signalling pathway. Biochem. J..

[16] Sorrell F., Szklarz M., Abdulazeez K., Elkins J., Knapp S. (2016). Family-wide Structural Analysis of Human Numb-Associated Protein Kinases. Structure.

[17] Eswaran J., Bernad A., Ligos J.M., Guinea B., Debreczeni J.É., Sobott F., Parker S., Najmanovich R., Turk B.E., Knapp S. (2008). Structure of the Human Protein Kinase MPSK1 Reveals an Atypical Activation Loop Architecture. Structure.

[18] Stairs D.B., Notarfrancesco K.L., Chodosh L.A. (2005). The serine/threonine kinase, Krct, affects endbud morphogenesis during murine mammary gland development. Transgenic Res..

[19] Guinea B., Ligos J.M., Lera T.L.D., Martín-Caballero J., Flores J., Peña M.G.D.L., García-Castro J., Bernada A. (2006). Nucleocytoplasmic shuttling of STK16 (PKL12), a Golgi-resident serine/threonine kinase involved in VEGF expression regulation. Exp. Cell Res..

[20] Ligos J.M., de Lera T.L., Hinderlich S., Guinea B., Sánchez L., Roca R., Valencia A., Bernad A. (2002). Functional interaction between the Ser/Thr kinase PKL12 and N-acetylglucosamine kinase, a prominent enzyme implicated in the salvage pathway for GlcNAc recycling. J. Biol. Chem..

[21] Liu J., Yang X., Li B., Wang J., Wang W., Liu J., Liu Q., Zhang X. (2017). STK16 regulates actin dynamics to control Golgi organization and cell cycle. Sci. Rep..

[22] Florante R., Rosa M., Surya C., Tattika S., Lisa O., Teli H., Maribeth S., Editte G. (2011). A genome-wide immunodetection screen in S. cerevisiae uncovers novel genes involved in lysosomal vacuole function and morphology. PLoS ONE.

[23] Hanks S.K., Hunter T. (1995). Protein kinases 6. The eukaryotic protein kinase superfamily: Kinase (catalytic) domain structure and classification. Faseb J..

[24] Chien C.T., Wang S., Rothenberg M., Jan L.Y., Jan Y.N. (1998). Numb-associated kinase interacts with the phosphotyrosine binding domain of Numb and antagonizes the function of Numb in vivo. Mol. Cell. Biol..

[25] Rhyu M.S., Jan L.Y., Jan Y.N. (1994). Asymmetric distribution of numb protein during division of the sensory organ precursor cell confers distinct fates to daughter cells. Cell.

[26] Uemura T., Shepherd S., Ackerman L., Jan L.Y., Jan Y.N. (1989). numb, a gene required in determination of cell fate during sensory organ formation in Drosophila embryos. Cell.

[27] Conner S.D., Schmid S.L. (2002). Identification of an adaptor-associated kinase, AAK1, as a regulator of clathrin-mediated endocytosis. J. Cell Biol..

[28] Conner S.D., Schröter T., Schmid S.L. (2010). AAK1-mediated micro2 phosphorylation is stimulated by assembled clathrin. Traffic.

[29] Neetu G.R., Sara O., Vannary M.Y., Sara H., Julien M., Jean-Christophe O.M., Alain I.L. (2011). The adaptor-associated kinase 1, AAK1, is a positive regulator of the Notch pathway. J. Biol. Chem..

[30] Sorensen E.B., Conner S.D. (2010). AAK1 regulates Numb function at an early step in clathrin-mediated endocytosis. Traffic.

[31] Krieger J.R., Paul T., Gajadhar A.S., Abhijit G., Moran M.F., C Jane M. (2013). Identification and selected reaction monitoring (SRM) quantification of endocytosis factors associated with Numb. Mol. Cell. Proteom..

[32] Lee D.-W., Zhao X., Zhang F., Eisenberg E., Greene L.E. (2005). Depletion of GAK/auxilin 2 inhibits receptor-mediated endocytosis and recruitment of both clathrin and clathrin adaptors. J. Cell Sci..

[33] Zhang C., Engqvist-Goldstein A., Carreno S., Owen D., Smythe E., Drubin D. (2010). Multiple roles for cyclin G-associated kinase in clathrin-mediated sorting events. Traffic.

[34] Kostich W., Hamman B.D., Li Y.W., Naidu S., Dandapani K., Feng J., Easton A., Bourin C., Baker K., Allen J. (2016). Inhibition of AAK1 Kinase as a Novel Therapeutic Approach to Treat Neuropathic Pain. J. Pharmacol. Exp. Ther..

[35] Abdel-Magid A.F. (2017). Inhibitors of Adaptor-Associated Kinase 1 (AAK1) May Treat Neuropathic Pain, Schizophrenia, Parkinson’s Disease, and Other Disorders. ACS Med. Chem. Lett..

[36] Bekerman E., Neveu G., Shulla A., Brannan J., Pu S.Y., Wang S., Xiao F., Barouch-Bentov R., Bakken R.R., Mateo R. (2017). Anticancer kinase inhibitors impair intracellular viral trafficking and exert broad-spectrum antiviral effects. J. Clin. Investig..

[37] Sona K., Lei C., Elena B., Gregory N., Rina B.B., Apirat C., Christina H., Michal Á., Steven D.J., Stefan K. (2015). Selective Inhibitors of Cyclin G Associated Kinase (GAK) as Anti-Hepatitis C Agents. J. Med. Chem..

[38] Pu S.Y., Wouters R., Schor S., Rozenski J., Barouch-Bentov R., Prugar L.I., Obrien C.M., Brannan J.M., Dye J.M., Herdewijn P. (2018). Optimization of isothiazolo[4,3-b]pyridine-based inhibitors of cyclin G associated kinase (GAK) with broad-spectrum antiviral activity. J. Med. Chem..

[39] Zhou H., Min X., Qian H., Gates A.T., Zhang X.D., Castle J.C., Stec E., Ferrer M., Strulovici B., Hazuda D.J. (2008). Genome-Scale RNAi Screen for Host Factors Required for HIV Replication. Cell Host Microbe.

[40] Liu F., Wang J., Yang X., Li B., Wu H., Qi S., Chen C., Liu X., Yu K., Wang W. (2016). Discovery of a Highly Selective STK16 Kinase Inhibitor. ACS Chem. Biol..

[41] Anderson A.M., Ragan M.A. (2016). Palmitoylation: A protein S-acylation with implications for breast cancer. NPJ Breast Cancer.

[42] Kumar S., Singh B., Dimmock J.R., Sharma R.K. (2011). N-myristoyltransferase in the leukocytic development processes. Cell Tissue Res..

[43] Udenwobele D.I., Su R.-C., Good S.V., Ball T.B., Varma Shrivastav S., Shrivastav A. (2017). Myristoylation: An important protein modification in the immune response. Front. Immunol..

[44] Resh M.D. (2013). Covalent lipid modifications of proteins. Curr. Biol. CB.

[45] Resh M.D. (2016). Fatty Acylation of Proteins: The Long and the Short of it. Prog. Lipid Res..

[46] Jiang H., Zhang X., Chen X., Aramsangtienchai P., Tong Z., Lin H. (2018). Protein Lipidation: Occurrence, Mechanisms, Biological Functions, and Enabling Technologies. Chem. Rev..

[47] Aicart-Ramos C., Valero R.A., Rodriguez-Crespo I. (2011). Protein palmitoylation and subcellular trafficking. BBA-Biomembr..

[48] Ryo M., Miki M., Takefumi U., Satoshi W., Eiji M., Naoyuki T., Michiyuki M., Tomohiko T. (2011). Palmitoylated Ras proteins traffic through recycling endosomes to the plasma membrane during exocytosis. Autophagy.

[49] Stoffel R.H., Randall R.R., Premont R.T., Lefkowitz R.J., Inglese J. (1994). Palmitoylation of G protein-coupled receptor kinase, GRK6. Lipid modification diversity in the GRK family. J. Biol. Chem..

[50] Konitsiotis A.D., Roßmannek L., Stanoev A., Schmick M., Pih B. (2017). Spatial cycles mediated by UNC119 solubilisation maintain Src family kinases plasma membrane localisation. Nat. Commun..

[51] Shenoy-Scaria A.M., Dietzen D.J., Kwong J., Link D.C., Lublin D.M. (1994). Cysteine3 of Src family protein tyrosine kinase determines palmitoylation and localization in caveolae. J. Cell Biol..

[52] Burgers P.P., Yuliang M., Luigi M., Mason M., Van Der Heyden M.A.G., Mark E., Arjen S., Taylor S.S., Heck A.J.R. (2012). A small novel A-kinase anchoring protein (AKAP) that localizes specifically protein kinase A-regulatory subunit I (PKA-RI) to the plasma membrane. J. Biol. Chem..

[53] Martin M.L., Busconi L. (2010). Membrane localization of a rice calcium-dependent protein kinase (CDPK) ismediated by myristoylation and palmitoylation. Plant J..

[54] Manandhar S.P., Florante R., Cocca S.M., Editte G. (2013). Saccharomyces cerevisiae Env7 is a novel serine/threonine kinase 16-related protein kinase and negatively regulates organelle fusion at the lysosomal vacuole. Mol. Cell. Biol..

[55] Manandhar S.P., Calle E.N., Editte G. (2014). Distinct palmitoylation events at the amino-terminal conserved cysteines of Env7 direct its stability, localization, and vacuolar fusion regulation in S. cerevisiae. J. Biol. Chem..

[56] Nolen B., Taylor S., Ghosh G. (2004). Regulation of Protein Kinases: Controlling Activity through Activation Segment Conformation. Mol. Cell.

[57] Natarajan K., Neuwald A.F. (2005). Did protein kinase regulatory mechanisms evolve through elaboration of a simple structural component?. J. Mol. Biol..

[58] Kohn A.D., Summers S.A., Birnbaum M.J., Roth R.A. (1996). Expression of a constitutively active Akt Ser/Thr kinase in 3T3-L1 adipocytes stimulates glucose uptake and glucose transporter 4 translocation. J. Biol. Chem..

[59] Xiao G., Sun S.C. (2000). Negative regulation of the nuclear factor κB-inducing kinase by a cis-acting domain. J. Biol. Chem..

[60] Liu J., Sudom A., Min X., Cao Z., Gao X., Ayres M., Lee F., Cao P., Yehnston S., Plotnikova O. (2012). Structure of the nuclear factor κB-inducing kinase (NIK) kinase domain reveals a constitutively active conformation. J. Biol. Chem..

[61] Thu Y.M., Richmond A. (2010). NF-κB inducing kinase: A key regulator in the immune system and in cancer. Cytokine Growth Factor Rev..

[62] Zheng W., Cai X., Li S., Li Z. (2018). Autophosphorylation mechanism of the Ser/Thr kinase Stk1 from Staphylococcus aureus. Front. Microbiol..

[63] Romano P.R., Garcia-Barrio M.T., Zhang X., Wang Q., Taylor D.R., Zhang F., Herring C., Mathews M.B., Qin J., Hinnebusch A.G. (1998). Autophosphorylation in the activation loop is required for full kinase activity in vivo of human and yeast eukaryotic initiation factor 2alpha kinases PKR and GCN2. Mol. Cell. Biol..

[64] Saul V.V., Laureano D.L.V., Maja M., Marcus K., Thomas B., Karin F.W., Katja B., M Lienhard S. (2013). HIPK2 kinase activity depends on cis-autophosphorylation of its activation loop. J. Mol. Cell Biol..

[65] Stoffel R.H., Inglese J., Macrae A.D., Lefkowitz R.J., Premont R.T. (1998). Palmitoylation increases the kinase activity of the G protein-coupled receptor kinase, GRK6. Biochemistry.

[66] Mei Y., Wang Y., Hu T., Yang X., Lozano-Duran R., Sunter G., Zhou X. (2018). Nucleocytoplasmic Shuttling of Geminivirus C4 Protein Mediated by Phosphorylation and Myristoylation Is Critical for Viral Pathogenicity. Mol. Plant.

[67] Kim J., Shishido T., Jiang X., Aderem A., Mclaughlin S. (1994). Phosphorylation, high ionic strength, and calmodulin reverse the binding of MARCKS to phospholipid vesicles. J. Biol. Chem..

[68] Ohmori S., Sakai N., Shirai Y., Yamamoto H., Miyamoto E., Shimizu N., Saito N. (2000). Importance of protein kinase C targeting for the phosphorylation of its substrate, myristoylated alanine-rich C-kinase substrate. J. Biol. Chem..

[69] Hata A., Chen Y.G. (2011). TGF-β Signaling from Receptors to Smads. Cold Spring Harb. Perspect. Biol..

[70] Macias M.J., Martin-Malpartida P., Massagué J. (2015). Structural determinants of Smad function in TGF-β signaling. Trends Biochem. Sci..

[71] Heldin C.H., Moustakas A. (2016). Signaling Receptors for TGF-β Family Members. Cold Spring Harb. Perspect. Biol..

[72] Zhang Y.E. (2017). Non-Smad Signaling Pathways of the TGF-β Family. Cold Spring Harb. Perspect. Biol..

[73] Massagué J. (1998). TGF-beta signal transduction. Annu. Rev. Biochem..

[74] Massagué J., Chen Y.G. (2000). Controlling TGF-β signaling. Genes Dev..

[75] Rik D., Zhang Y.E. (2003). Smad-dependent and Smad-independent pathways in TGF-beta family signalling. Nature.

[76] Shi Y., Massague J. (2003). Mechanisms of tgf-Beta signaling from cell membrane to the nucleus. Cell.

[77] Travis M.A., Sheppard D. (2014). TGF-beta activation and function in immunity. Annu. Rev. Immunol..

[78] Katz L.H., Ying L., Jiun-Sheng C., MuñOz N.M., Avijit M., Jian C., Lopa M. (2013). Targeting TGF-β signaling in cancer. Expert Opin. Ther. Targets.

[79] Meng X.M., DJ N., Lan H.Y. (2016). TGF-β: The master regulator of fibrosis. Nat. Rev. Nephrol..

[80] Ulrika B., Stefan K. (2015). TGF-β signaling in the control of hematopoietic stem cells. Blood.

[81] Li J., Wang Y., Meng X., Liang H. (2018). Modulation of transcriptional activity in brain lower grade glioma by alternative splicing. PeerJ.

[82] Park J.T., Uehara T. (2008). How Bacteria Consume Their Own Exoskeletons (Turnover and Recycling of Cell Wall Peptidoglycan). Microbiol. Mol. Biol. Rev..

[83] Lenardon M.D., Munro C.A., Gow N.A. (2010). Chitin synthesis and fungal pathogenesis. Curr. Opin. Microbiol..

[84] Moussian B. (2008). The role of GlcNAc in formation and function of extracellular matrices. Comp. Biochem. Physiol. B Biochem. Mol. Biol..

[85] Dennis J.W., Nabi I.R., Demetriou M. (2009). Metabolism, Cell Surface Organization, and Disease. Cell.

[86] Konopka J.B. (2012). N-acetylglucosamine (GlcNAc) functions in cell signaling. Scientifica (Cairo).

[87] Wolosker H., Kline D., Bian Y., Blackshaw S., Cameron A.M., Fralich T.J., Schnaar R.L., Snyder S.H. (1998). Molecularly cloned mammalian glucosamine-6-phosphate deaminase localizes to transporting epithelium and lacks oscillin activity. FASEB J..

[88] Reutter W., Stäsche R., Stehling P., Baum O., Hans-Joachim G., Sigrun G. (1997). The Biology of Sialic Acids: Insights into their Structure, Metabolism and Function in Particular during Viral Infection. Glycosciences: Status and Perspectives.

[89] Bond M.R., Hanover J.A. (2015). A little sugar goes a long way: The cell biology of O-GlcNAc. J. Cell Biol..

[90] Corfield A.P., Berry M. (2015). Glycan variation and evolution in the eukaryotes. Trends Biochem. Sci..

[91] Shamoon N., Angelo G., Esteban A., Konopka J.B. (2011). N-acetylglucosamine (GlcNAc) induction of hyphal morphogenesis and transcriptional responses in Candida albicans are not dependent on its metabolism. J. Biol. Chem..

[92] Thomas N., Joanne H., Mcconville M.J. (2010). Evidence that intracellular stages of Leishmania major utilize amino sugars as a major carbon source. PLoS Pathog..

[93] Hyunsook L., Sun-Jung C., Il Soo M. (2014). The non-canonical effect of N-acetyl-D-glucosamine kinase on the formation of neuronal dendrites. Mol. Cells.

[94] Islam M.A., Sharif S.R., Lee H.S., Moon I.S. (2015). N-Acetyl-D-Glucosamine Kinase Promotes the Axonal Growth of Developing Neurons. Mol. Cells.

[95] Islam M.A., Sharif S.R., Lee H.S., Seog D.H., Moon I.S. (2015). N-acetyl-D-glucosamine kinase interacts with dynein light-chain roadblock type 1 at Golgi outposts in neuronal dendritic branch points. Exp. Mol. Med..

[96] Sharif S.R., Islam A., Moon I.S. (2016). N-Acetyl-D-Glucosamine Kinase Interacts with Dynein-Lis1-NudE1 Complex and Regulates Cell Division. Mol. Cells.

[97] Sharif S.R., Lee H., Islam M.A., Seog D.H., Moon I.S. (2015). N-acetyl-D-glucosamine kinase is a component of nuclear speckles and paraspeckles. Mol. Cells.

[98] Wilson S.H., Bailey A.M., Nourse C.R., Mattei M.G., Byrne J.A. (2001). Identification of MAL2, a novel member of the mal proteolipid family, though interactions with TPD52-like proteins in the yeast two-hybrid system. Genomics.

[99] De Marco M.C., Puertollano R., Martínezmenárguez J.A., Alonso M.A. (2010). Dynamics of MAL2 during glycosylphosphatidylinositol-anchored protein transcytotic transport to the apical surface of hepatoma HepG2 cells. Traffic.

[100] In J.G., Tuma P.L. (2010). MAL2 selectively regulates polymeric IgA receptor delivery from the Golgi to the plasma membrane in WIF-B cells. Traffic.

[101] Marco M.C., De Fernando M.B., Leonor K., Albar J.P., Isabel C., Vaerman J.P., Mónica M., Byrne J.A., Alonso M.A. (2002). MAL2, a novel raft protein of the MAL family, is an essential component of the machinery for transcytosis in hepatoma HepG2 cells. J. Cell Biol..

[102] In J.G., Striz A.C., Antonio B., Tuma P.L. (2014). Serine/threonine kinase 16 and MAL2 regulate constitutive secretion of soluble cargo in hepatic cells. Biochem. J..

[103] Madrid R., Aranda J.F., Rodríguez-Fraticelli A.E., Ventimiglia L., Andrés-Delgado L., Shehata M., Fanayan S., Shahheydari H., Gómez S., Jiménez A. (2010). The Formin INF2 Regulates Basolateral-to-Apical Transcytosis and Lumen Formation in Association with Cdc42 and MAL2. Dev. Cell.

[104] Rao K.H., Ghosh S., Datta A. (2016). Env7p Associates with the Golgin Protein Imh1 at thetrans-Golgi Network inCandida albicans. MSphere.

[105] Alfonso L.C., Striz A.C., Tuma P.L. (2018). A Serine/Threonine Kinase 16-Based Phospho-Proteomics Screen Identifies WD Repeat Protein-1 as a Regulator of Constitutive Secretion. Sci. Rep..

[106] Amberg D.C., Basart E., Botstein D. (1995). Defining protein interactions with yeast actin in vivo. Nat. Struct. Biol..

[107] Rodal A.A., Tetreault J.W., Lappalainen P., Drubin D.G., Amberg D.C. (1999). Aip1p interacts with cofilin to disassemble actin filaments. J. Cell Biol..

[108] Shoichiro O. (2003). Regulation of actin filament dynamics by actin depolymerizing factor/cofilin and actin-interacting protein 1: New blades for twisted filaments. Biochemistry.

[109] Gissen P., Arias I.M. (2015). Structural and functional hepatocyte polarity and liver disease. J. Hepatol..

[110] Zeigerer A., Wuttke A., Marsico G., Seifert S., Kalaidzidis Y., Zerial M. (2017). Functional properties of hepatocytes in vitro are correlated with cell polarity maintenance. Exp. Cell Res..

[111] Li B., Trueb B. (2000). DRG represents a family of two closely related GTP-binding proteins. BBA-Gene Struct. Expr..

[112] Sazuka T., Kinoshita M., Tomooka Y., Ikawa Y., Noda M., Kumar S. (1992). Expression of DRG during murine embryonic development. Biochem. Biophys. Res. Commun..

[113] Sazuka T., Tomooka Y., Ikawa Y., Noda M., Kumar S. (1992). DRG: A novel developmentally regulated GTP-binding protein. Biochem. Biophys. Res. Commun..

[114] Mahajan M.A., Park S.T., Sun X.H. (1996). Association of a novel GTP binding protein, DRG, with TAL oncogenic proteins. Oncogene.

[115] Zhao X.F., Aplan P.D. (1998). SCL binds the human homologue of DRG in vivo 1. Biochim. Biophys. Acta (BBA)-Mol. Cell Res..

[116] Ishikawa K., Azuma S., Ikawa S., Morishita Y., Gohda J., Akiyama T., Semba K., Inoue J.I. (2003). Cloning and characterization of Xenopus laevis drg2, a member of the developmentally regulated GTP-binding protein subfamily. Gene.

[117] Schellhaus A.K., Morenoandrés D., Chugh M., Yokoyama H., Moschopoulou A., De S., Bono F., Hipp K., Schäffer E., Antonin W. (2017). Developmentally Regulated GTP binding protein 1 (DRG1) controls microtubule dynamics. Sci. Rep..

[118] Dang T., Jang S.H., Back S.H., Park J.W., Han I.S. (2018). DRG2 Deficiency Causes Impaired Microtubule Dynamics in HeLa Cells. Mol. Cells.

[119] Huang S., Wang Y. (2017). Golgi structure formation, function, and post-translational modifications in mammalian cells. F1000Research.

[120] Egea G., Lázaro-Diéguez F., Vilella M. (2006). Actin dynamics at the Golgi complex in mammalian cells. Curr. Opin. Cell Biol..

[121] Egea G., Serra-Peinado C., Salcedo-Sicilia L., Gutiérrez-Martínez E. (2013). Actin acting at the Golgi. Histochem. Cell Biol..

[122] Vinke F.P., Grieve A.G., Rabouille C. (2011). The multiple facets of the Golgi reassembly stacking proteins. Biochem. J..

[123] Barr F.A., Puype M., Vandekerckhove J., Warren G. (1997). GRASP65, a protein involved in the stacking of Golgi cisternae. Cell.

[124] Tang D., Zhang X., Huang S., Yuan H., Li J., Wang Y. (2016). Mena-GRASP65 interaction couples actin polymerization to Golgi ribbon linking. Mol. Biol. Cell.

[125] Romina Ines C., Raffaella B., Maria Luisa B., Daniela S., Inmaculada A., Nobuhiro N., Daniela C., Antonino C. (2015). JNK2 controls fragmentation of the Golgi complex and the G2/M transition through phosphorylation of GRASP65. J. Cell Sci..

[126] Feinstein T.N., Linstedt A.D. (2008). GRASP55 regulates Golgi ribbon formation. Mol. Biol. Cell.

[127] Valente C., Colanzi A. (2015). Mechanisms and Regulation of the Mitotic Inheritance of the Golgi Complex. Front. Cell Dev. Biol..

[128] Champion L., Linder M.I., Kutay U. (2016). Cellular Reorganization during Mitotic Entry. Trends Cell Biol..

[129] Lowe M. (2011). Structural organization of the Golgi apparatus. Curr. Opin. Cell Biol..

[130] Lin C.M., Chen H.J., Leung C.L., Parry D.A., Liem R.K. (2005). Microtubule actin crosslinking factor 1b: A novel plakin that localizes to the Golgi complex. J. Cell Sci..

[131] Wei J.H., Seemann J. (2017). Golgi ribbon disassembly during mitosis, differentiation and disease progression. Curr. Opin. Cell Biol..

